# Leptomeningeal metastases in patients with human epidermal growth factor receptor 2 positive breast cancer: Real‐world data from a multicentric European cohort

**DOI:** 10.1002/ijc.34135

**Published:** 2022-06-25

**Authors:** Ivica Ratosa, Nika Dobnikar, Michele Bottosso, Maria Vittoria Dieci, William Jacot, Stéphane Pouderoux, Domen Ribnikar, Léa Sinoquet, Valentina Guarneri, Tanja Znidaric, Amélie Darlix, Gaia Griguolo

**Affiliations:** ^1^ Division of Radiotherapy Institute of Oncology Ljubljana Ljubljana Slovenia; ^2^ Faculty of Medicine University of Ljubljana Ljubljana Slovenia; ^3^ Division of Oncology 2 Istituto Oncologico Veneto IRCCS Padova Italy; ^4^ Department of Surgery, Oncology and Gastroenterology University of Padova Padova Italy; ^5^ Department of Medical Oncology, Institut régional du Cancer de Montpellier University of Montpellier Montpellier France; ^6^ Division of Medical Oncology Institute of Oncology Ljubljana Ljubljana Slovenia; ^7^ Department of Oncology University Medical Centre Maribor Maribor Slovenia; ^8^ Institut de Génomique Fonctionnelle, INSERM, CNRS University of Montpellier Montpellier France

**Keywords:** anti‐HER2 therapy, breast cancer, human epidermal growth factor receptor 2, leptomeningeal metastases, radiation therapy

## Abstract

In patients with human epidermal growth factor receptor 2 positive (HER2+) breast cancer, leptomeningeal metastases (LM) are a rare but often a fatal clinical scenario. In this multicentric study, clinical and pathologic characteristics of patients with HER2+ breast cancer developing LM were described, as well as survival outcomes. Data were gathered retrospectively from medical records of 82 patients with advanced HER2+ breast cancer and LM treated between August 2005 and July 2020. Following LM diagnosis, 79 (96.3%) patients received at least one line of anti‐HER2 therapy, 25 (30.5%) patients received intrathecal therapy and 58 (70.7%) patients received radiotherapy. Overall survival (OS) was 8.3 months (95% confidence interval [CI] 5.7‐11), 1‐year OS was 42%, and 2‐year OS was 21%. At univariate analysis, patients who were treated after 2010, had better Karnofsky performance status, were free of neurological symptoms, had better prognostic, received chemotherapy (OS difference 9.4 months, *P* = .024), or monoclonal antibodies (trastuzumab ± pertuzumab; OS difference 6.1 months; *P* = .013) after LM diagnosis, had a statistically significantly longer OS. Presence of neurological symptoms (hazard ratio 3.32, 95% CI 1.26‐8.73; *P* = .015) and not having received radiotherapy (hazard ratio 2.02, 95% CI 1.09‐3.72; *P* = .024) were all associated with poorer OS at multivariate analysis. To summarize, not having neurological symptoms and receiving RT at LM diagnosis were associated with prolonged OS in our cohort. Survival seemed to be prolonged with multimodality treatment, which included targeted therapy, chemotherapy, and RT to the LM sites.

## What's new?

Patients with human epidermal growth factor receptor 2‐positive (HER2+) breast cancer and leptomeningeal metastasis (LM) are likely to suffer neurological damage. The degree to which neurological symptoms are evident and their impact on survival remain uncertain. Here, investigation of pathological features and survival in a European cohort of HER2+ breast cancer patients with LM shows that preexisting brain metastases are common at LM diagnosis, affecting as many as two‐thirds of patients. Overall survival was longer among patients who lacked neurological symptoms and who received radiotherapy at LM diagnosis. Longer survival was further linked to the use of multimodality therapy.

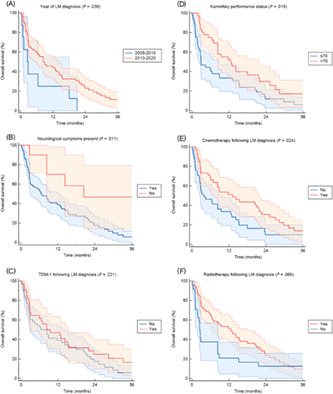

**Abbreviations**
ASCO/CAP: American Society of Clinical Oncology/College of American Pathologists
B‐GPA: breast‐graded prognostic assessment
CI: confidence intervals
CNS: central nervous system
CNS‐PD: central nervous system progressive disease
ER: estrogen receptor
FISH: fluorescent in situ hybridization
G: histological grade
HER2+: human epidermal growth receptor 2 positive
HR: hormonal receptor
IHC: immunohistochemistry
IT: intrathecal therapy
KPS: Karnofsky performance status
LM: leptomeningeal metastases
MBC: metastatic breast cancer
MRI: magnetic resonance imaging
n: number
NA: not available
OS: overall survival
PR: progesterone receptor
RT: radiation therapy
SS‐BM: simple survival score
TDM‐1: trastuzumab‐emtansine

## 1 INTRODUCTION

Leptomeningeal metastases (LM) are a complication of relatively uncommon occurrence in patients with breast cancer. The presence of LM usually alters the course of the metastatic disease, resulting in a bleak prognosis and a reduction in patients' quality of life. The estimated incidence of LM in unselected patients with breast cancer is very low, appearing to be less than 1%, but the percentage increases in patients with advanced breast cancer, reaching 6.6% in clinical studies^1^, ^2^ and up to 16% in autopsy series.^3^, ^4^

Breast cancer tumor biology may play a role in the incidence of LM, time between primary breast cancer diagnosis and LM diagnosis, and subsequent overall survival (OS). The distribution of primary breast cancer surrogate clinical subtypes in LM patients varies significantly. Human epidermal growth receptor 2 positive (HER2+) breast cancer cells may be more neurotropic than other breast cancer cell populations,^5^ and HER2+ breast cancer cells have a high proclivity to spread to the brain.^6^ The propensity of HER2‐positive breast cancer cells to metastasize to the brain might be explained by several genetic and molecular pathways. HER2‐positive BC subtypes are more likely to have X‐inactive‐specific transcript (XIST) downregulation, which promotes epithelial‐mesenchymal transition, motility, and migration of primary BC cells and especially increases the proclivity of circulating tumor cells (CTCs) to invade the brain.^7^ CTC passing through the blood‐brain barrier and brain colonization are further aided by the prometastatic actions of β4 integrin, to which HER2‐positive cells are especially susceptible. The β4 integrin promotes the adherence of breast tumor cells to microvascular endothelial cells and interacts with the HER2 receptor. In response to β4 signaling, HER2‐positive breast tumor cells release increased vascular endothelial growth factor (VEGF). VEGF impairs the integrity of endothelial tight and adherence junctions, allowing tumor cells to attach and cross the blood‐brain barrier. VEGF increases vascular growth once tumor cells have infiltrated the brain.^8^, ^9^

However, breast cancer subtypes appear to have a different natural propensity to metastasis to the leptomeninges than brain parenchyma.^10^ In fact, invasive lobular breast cancer (mostly luminal‐A‐like subtype) has been shown to be more likely to spread to the leptomeninges than other histological types.^11^ Hormone receptor positive (HR+)/HER2− tumors are the most common breast cancer subtypes causing symptomatic LM (44%‐71.4%), followed by HR−/HER2− (12.8%‐25.5%), HR+/HER2+ (10%‐18%), and finally HR−/HER2+ (0.3%‐8.5%).^10^, ^12^, ^13^, ^14^, ^15^ Nonetheless, data is inconclusive, as a few observational studies reported the highest proportion of LM among patients with HR−/HER2− tumors (up to 35%).^16^ On the other hand, LM are becoming more common in patients with HER2+ subtypes of breast cancer, possibly as a result of advances in systemic anti‐HER2 therapy over the last decade, which have resulted in prolonged metastatic survival and better systemic disease control.^14^

Treatment modalities for LM are usually not supported by data from randomized clinical trials and are often based only on expert opinions.^17^ Available treatment options include standard systemic chemotherapy, targeted (anti‐HER2) therapy, endocrine therapy, intrathecal therapy (IT), and radiation therapy (RT).^18^ For patients with HER2+ metastatic disease the treatment landscape is constantly changing, and patient subgroups with specific clinical needs, such as those with central nervous system (CNS) involvement, are emerging.^19^ Several anti‐HER2 targeted agent, including trastuzumab, pertuzumab, trastuzumab‐emtansine (TDM‐1), lapatinib, neratinib, trastuzumab deruxtecan, and tucatinib, have demonstrated antitumor activity in the brain by extending time‐to‐brain metastases development and time‐to‐brain progression in patients with brain metastases.^20^, ^21^, ^22^, ^23^, ^24^, ^25^ However, due to the rarity of this complication, little is known regarding the clinical outcomes of patients with HER2‐positive breast cancer and LM. The purpose of our study is to characterize clinical and pathologic features, as well as survival outcomes, of patients with HER2+ breast cancer‐related LM using real‐world data from a multicentric retrospective cohort.

## 2 METHODS

### 2.1 Study design and data collection

**FIGURE 1:**
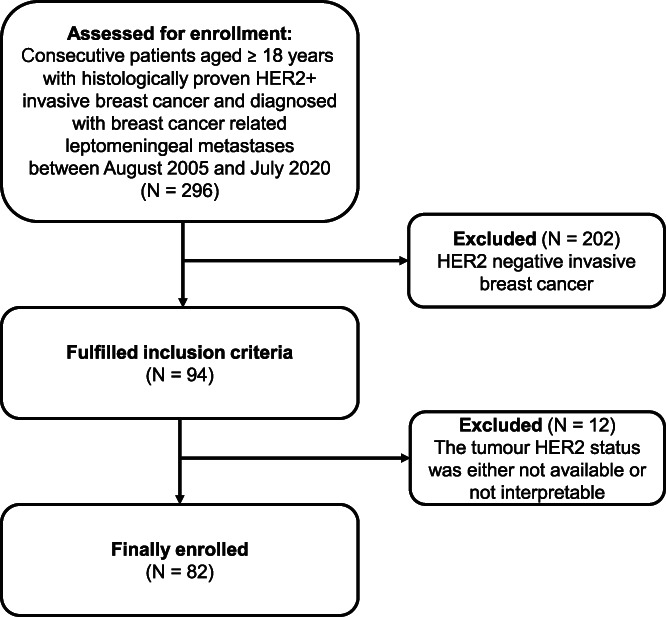
Flow diagram of the patients included in our study. HER2+, human epidermal growth receptor 2 positive; N, number

Patients' clinical and histopathological characteristics, local and systemic treatments data were retrieved from medical records for patients, aged ≥18 years with histologically proven invasive HER2+ breast cancer and diagnosis of LM, with or without brain metastases. The flow diagram for patients included in our study is shown in Figure 1. Data were retrospectively collected, and the study cut‐off date was March 31, 2021. LM were diagnosed by magnetic resonance imaging (MRI), combined with neurological signs or positive cytology performed on cerebrospinal fluid, as by guidelines (ESMO‐EANO). For each patient three different prognostic indices were calculated as defined in the literature: *breast‐graded prognostic assessment* (B‐GPA),^26^ *the simple survival score* (SS‐BM),^27^ and *INDEX score*, which integrates patients' clinical characteristics, breast cancer biological subtype and LM treatment characteristics.^28^ De novo metastatic breast cancer was defined as metastatic disease diagnosed by diagnostic imaging up to 3 months following initial breast cancer pathohistological diagnosis. According to the American Joint Committee on Cancer's Staging System for Breast Cancer, Eighth Edition,^29^ and as described in the most recent research,^30^, ^31^ recurrent breast cancer was classified as breast cancer that recurred 3 months or later after the first breast cancer diagnosis. The neurologic symptoms associated with LM included but were not limited to cranial nerves symptoms (eg, double vision, difficulty swallowing or speaking), headaches, nausea, impaired coordination in the arms or legs, or numbness. The emergence of new CNS metastasis or progression of existing CNS metastasis after the first treatment was defined as CNS progressive disease (CNS‐PD). CNS progression included the progression of preexisting brain lesions (both LM or brain metastases) or the development of new CNS lesions based on radiologic imaging criteria. All included patients were at least 18 years old and were diagnosed with LM between August 2005 and July 2020.

### 2.2 Identification of breast cancer subtypes

Molecular breast cancer subtypes were identified as HR+/HER2+ (estrogen receptor positivity, ER+; HER2+; any progesterone receptor [PR] expression; any Ki67), and HR−/HER2+ (HER2+, PR−, ER−). Routine diagnostic pathology reports were used to assess the ER, PR, and HER2 receptor status of primary breast cancer tumors or metastatic disease at the time of diagnosis and were evaluated according to then valid American Society of Clinical Oncology/College of American Pathologists (ASCO/CAP) recommendations. Hormone receptor‐positive disease was classified as ER+ and/or PR+ disease with a cut off value of 1%. The status of ER or PR were determined using standard immunohistochemistry (IHC) methods as by clinical practice of the participating centers (see Supplementary Materials and Methods for more details).

### 2.3 Statistical analysis

**FIGURE 2:**
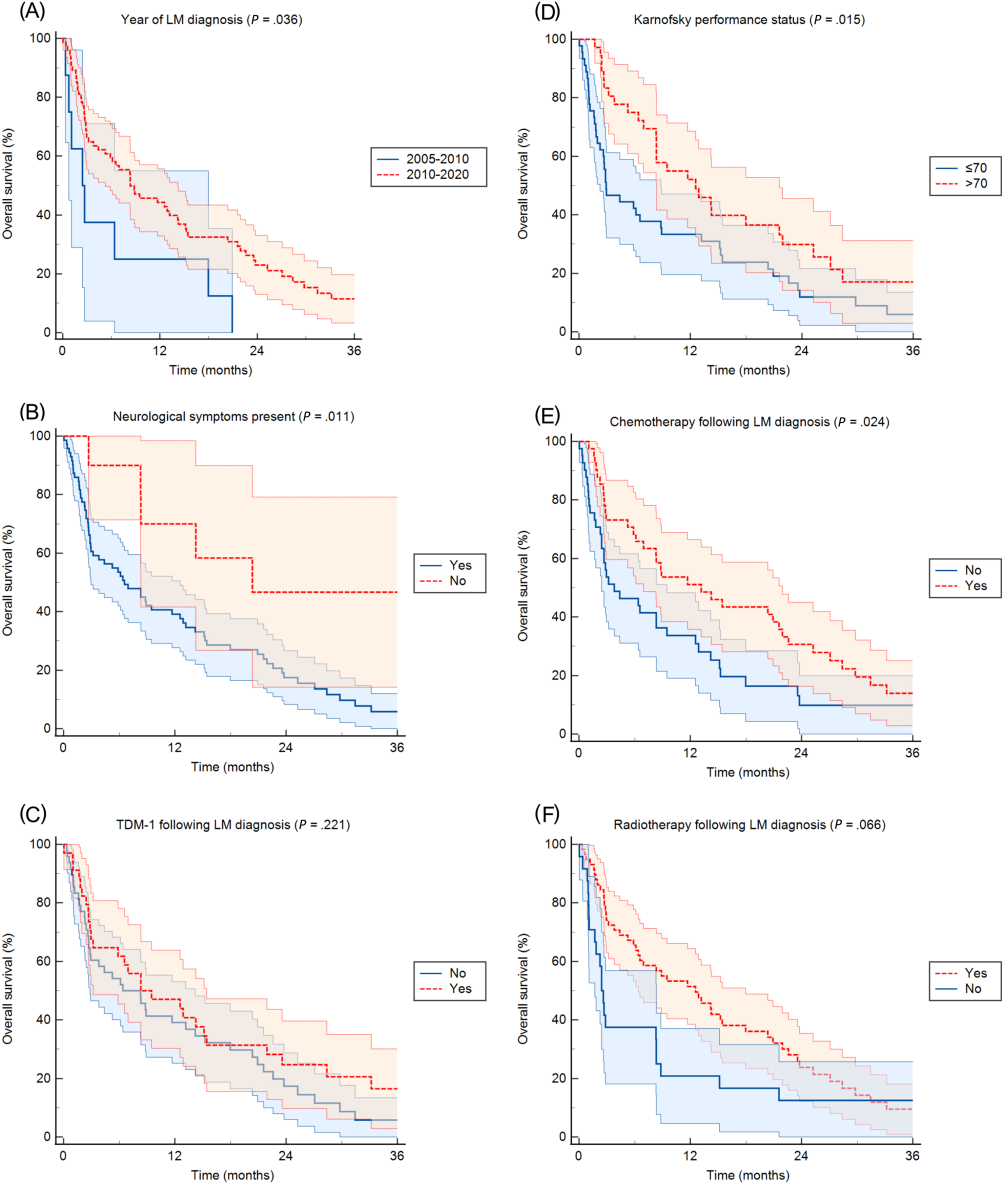
Patient Kaplan‐Meier curves of OS according to year of leptomeningeal metastases diagnosis (A), the presence or absence of neurological symptoms (B), the use of trastuzumab‐emtansine following LM diagnosis (C), the patients' performance status (D), the use of chemotherapy (E) and of radiation therapy (F). LM, leptomeningeal metastases; TDM‐1, trastuzumab‐emtansine [Color figure can be viewed at wileyonlinelibrary.com]

Data were analyzed using IBM SPSS Statistics software version 26 (Statistical package for the Social Sciences Statistical Software, SPSS Inc, IBM Corporation, Armonk, New York). A Pearson's chi‐square test was used to compare categorical variables between two groups. The Kaplan‐Meier method was used to calculate estimated survival curves, and the log‐rank test was used to compare two groups. Cox's proportional hazards model was used to calculate additional covariates. To account for variable interactions, we used less stringent *P* values (*P* < .10) obtained in the univariate analysis to test the variables in the multivariate analysis. To avoid obscuring or confounding the relationship between the independent and dependent variables, prognostic scores were excluded from the multivariate analysis. Overall survival (OS) was defined as time interval from LM diagnosis to date of death or last follow‐up. The binary multivariate logistic regression method was used to identify factors that were significantly associated with the progression of intracranial disease. Statistical analysis also included descriptive statistics. Data were expressed as median with a range for continuous variables, and as counts with frequencies for categorical data. All tests were two‐sided, and the statistical significance level was set at *P* = .05. The effect sizes [given as hazard ratios with 95% confidence intervals (CI)] were calculated using univariate and multivariate logistic regressions. Figure 2 was created using MedCalc Statistical Software version 20.103 (MedCalc Software bv, Ostend, Belgium; https://www.medcalc.org; 2020).

## 3 RESULTS

### 3.1 Patient characteristics

In total, 82 patients with HER2+ primary breast cancer and LM were included in the study. Median age at breast cancer diagnosis was 59 years (range, 41‐74) and median age at LM diagnosis was 64 years (range, 48‐76). Median time from primary breast cancer diagnosis to LM development was 52.8 months (range, 0.6‐188.2) for HR+/HER2+ breast cancer subtype and 43.2 months (range, 9.1‐244.4) for HR−/HER2+ breast cancer subtype (*P* = .852). The median time from metastatic disease to LM diagnosis was 21.1 months (range, 0‐128.3). Imaging alone (n = 55, 67.1%), imaging and cerebrospinal fluid cytology (n = 14, 17.1%), and cerebrospinal fluid cytology alone (n = 13, 15.9%) were used to diagnose LM.

At the time of LM diagnosis, two‐thirds of patients (n = 54, 64.6%) also had been diagnosed with concurrent brain metastases, while 29 (35.4%) only had LM. When brain parenchymal metastases were diagnosed in patients with LM, they were found concurrently in 26 (31.7%) patients, before LM diagnosis in 25 (30.5%) patients and after LM diagnosis in 2 (2.4%) patients. Patients' clinical and pathohistological characteristics are summarized in Table 1.

**TABLE 1:** Patients' clinical and pathohistological characteristics

Characteristic	n (%)
Histology	
Invasive ductal carcinoma	72 (87.8)
Invasive lobular carcinoma	7 (8.5)
Mixed (invasive lobular and ductal carcinoma)	2 (2.4)
Invasive apocrine carcinoma	1 (1.2)
Histological grade	
G1	2 (2.4)
G2	23 (28)
G3	48 (58.6)
Unknown	9 (11.0)
Hormonal receptors	
HR+	49 (59.8)
HR−	33 (40.2)
Metastatic disease	
De novo metastatic breast cancer	20 (24.4)
Recurrent breast cancer	62 (75.6)
Year of LM diagnosis	
2005‐2009	8 (9.8)
2010‐2020	74 (90.2)
Karnofsky PS at LM diagnosis	
100	1 (3.7)
80‐90	33 (40.2)
60‐70	18 (21.9)
≤50	27 (33.0)
Unknown	1 (1.2)
Extracranial disease at LM diagnosis	
No	5 (6.1)
Yes	77 (93.9)
Bone only	7 (8.5)
Visceral and bone (lung, liver, other)	44 (53.7)
Visceral only (lung, liver, other)	26 (31.7)
MRI findings at LM diagnosis	
Focal dural enhancement	23 (28.0)
Diffuse dural enhancement	32 (39.0)
Unknown/not reported or MRI not performed	27 (33.0)
LM present in other parts of neuroaxis	
Yes	10 (12.2)
No	56 (68.3)
Unknown	16 (19.5)
Hydrocephalus at LM diagnosis	
Yes	6 (7.3)
No	62 (75.6)
Unknown	14 (17.1)
B‐GPA at LM diagnosis	
Score 2	23 (28)
Score 3	49 (59.8)
Score 4	10 (12.2)
Simple survival score (SS‐BM) at LM diagnosis	
Score A	34 (41.5)
Score B	46 (56.1)
Score C	2 (2.4)
INDEX score at LM diagnosis	
Score A	31 (37.8)
Score B	15 (18.3)
Score C	25 (30.5)
Score D	11 (13.4)

Abbreviations: B‐GPA, breast‐graded prognostic assessment; G, histological grade; HER2+, human epidermal growth receptor 2 positive; HR, hormonal receptors, PS, performance status according to The Eastern Cooperative Oncology Group (ECOG) score; HR+, hormone receptor positive; LM, leptomeningeal metastases; MRI, magnetic resonance imaging; n, number.

Hormonal receptor status (HR+ or HR−) was known for 81 (98.8%) patients. Detailed data on systemic treatment received for metastatic disease prior to LM diagnosis is presented in Table S1. Prior to LM diagnosis, 9 (10.1%) patients received brain RT and 15 (18.1%) brain surgery due to brain metastases.

Data regarding treatment received after LM diagnosis are summarized in Table 2. Following LM diagnosis, 79 (96.3%) patients received at least one line of anti‐HER2 therapy. Eleven (13.4%) patients received three modalities of systemic treatment (chemotherapy, endocrine therapy, and anti‐HER2 therapy), 30 (36.6%) patients received chemotherapy and anti‐HER2 therapy, 4 (4.9%) patients received endocrine therapy and anti‐HER2 therapy, 34 (41.5%) patients received anti‐HER2 therapy alone, and 3 (3.7%) patients received no systemic treatment. Sixty‐seven (81.7%) patients were treated with trastuzumab‐based therapy; either alone (n = 41, 50%), in combination with pertuzumab (n = 24, 29.3%) or in combination with lapatinib (n = 6, 7.3%). Following LM diagnosis, 34 (41.5%) patients received TDM‐1 and 26 (31.1%) patients received lapatinib. Sixteen (19.5%) patients received a combination of lapatinib and capecitabine.

**TABLE 2:** Systemic and local treatment modalities following LM diagnosis

Treatment modality	All patients (n = 82, 100%)	HR+ (n = 49, 100%)	HR− (n = 33, 100%)	*P* value
Steroid therapy				.088
No	14 (17.1)	6 (12.2)	8 (24.2)	
Yes	68 (82.9)	43 (87.8)	25 (75.8)	
Endocrine therapy				.002
0	67 (81.7)	34 (69.4)	33 (100%)	
1 line	11 (13.4)	11 (22.4)	0	
≥2 lines	4 (4.9)	4 (8.2)	0	
Chemotherapy				.505
0	41 (50.0)	23 (46.9)	18 (54.5)	
1 line	31 (37.8)	17 (34.7)	14 (42.4)	
≥2 line	10 (12.2)	9 (18.4)	1 (3.4)	
HER2 targeted therapy^a^				.408
0	3 (3.7)	3 (6.1%)	0	
1 line	71 (86.6)	41 (83.7)	30 (90.9)	
≥2 lines	8 (9.7)	5 (10.2)	3 (9.1)	
Monoclonal antibody				.178
Trastuzumab	41 (50.0)	23 (46.9)	18 (54.5)	
Trastuzumab and pertuzumab	24 (29.3)	18 (36.7)	6 (18.2)	
No	15 (18.3)	6 (12.2)	9 (27.3)	
Unknown	2 (2.4)	2 (4.1)	0	
TDM‐1‐based therapy				.103
Yes	34 (41.5)	18 (36.7)	16 (48.5)	
No	43 (52.4)	26 (53.1)	17 (51.5)	
Unknown	5 (6.1)	5 (10.2)	0	
Lapatinib‐based therapy				.561
Yes	26 (31.7)	12 (24.5)	14 (42.4)	
No	54 (65.9)	35 (71.4)	19 (57.6)	
Unknown	2 (2.4)	2 (4.1)	0	
Intrathecal therapy (any)				.552
Yes	25 (30.5)	15 (30.6)	10 (30.3)	
No	57 (69.5)	34 (69.4)	23 (69.7)	
CNS RT after LM diagnosis				.173
Yes (first RT course, RT‐*naive*)	45 (54.8)	24 (49.0)	21 (63.6)	
Yes (second RT course, reirradiation)^b^	13 (15.9)	7 (14.3)	6 (18.2)	
No RT	24 (29.3)	18 (36.7)	6 (18.2)	
Type of CNS RT received after LM diagnosis				.201
WBRT	41 (50.0)	20 (40.8)	21 (63.6)	
Partial brain RT	6 (7.3)	2 (4.1)	4 (12.1)	
SRS	8 (9.8)	6 (12.2)	2 (6.1)	
Spinal RT	3 (3.7)	3 (6.1)	0	

Abbreviations: CNS, central nervous system; HR, hormonal receptors; LM, leptomeningeal metastases; n, number; RT, radiation therapy; SRS, stereotactic radiosurgery; TDM‐1, trastuzumab emtansine; WBRT, whole brain radiation therapy.

^a^ Targeted therapy, including trastuzumab, pertuzumab, trastuzumab emtansine, lapatinib.

^b^ Patients were previously treated with radiation therapy for brain parenchymal metastases (n = 9) or due to LM (n = 5).

Of the 25 patients who received IT, 22 (88.0%) received methotrexate, 1 patient received cytarabine, 1 patient received trastuzumab‐only, and 1 patient received methotrexate, cytarabine, and trastuzumab. With the exception of endocrine treatment (*P* = .002), we found no statistically significant differences in the kind of systemic or local treatment administered based on breast cancer subtype (Table 2).

### 3.2 Outcome

The median follow‐up from time of LM diagnosis was 8.3 months (range, 0.1‐77.3). The data for the emergence of new CNS metastasis or progression of existing CNS metastasis was available for 64 patients. CNS‐PD was observed in 57 (89.1%) during the follow‐up period. Out of 45 evaluable patients receiving RT, 39 (86.7%) developed CNS‐PD compared to patients who did not receive RT (n = 18/19, 94.7%), and the difference was not statistically significant (*P* = .664). None of the factors tested in the binary multivariate logistic regression model, including HR status (*P* = .550), de novo vs recurrent disease (*P* = .611), the presence of brain metastases (*P* = .162), the receipt of chemotherapy (*P* = .999), TDM‐1 (*P* = .828), or lapatinib therapy (*P* = .551), or local treatments such as brain metastases surgery (*P* = .999), IT (*P* = .871), and RT (*P* = .362), appeared to be significantly associated with intracranial disease progression. Following RT, we observed no improvement in clinical symptoms of LM in 22 (37.9%), improvement in 17 (29.3%), and an unknown clinical response in 19 (32.8%) patients.

At the time of data cut‐off, 71 (86.6%) patients had died. Fifty‐two (63.4%) patients died due to progression of the intracranial disease, 14 (17.1%) patients died due to extracranial disease progression, while the cause of death was not clearly identifiable from medical records for 16 (19.5%) patients.

Median OS from LM diagnosis in the study cohort was 8.3 months (95% CI 5.7‐11*x*). The estimated OS rate was 42% at 1 year, 21% at 2 years, and 10% at 3 years, respectively. The impact of several known prognostic factors on OS was investigated using univariate Cox regression (Table 3). No significant difference in observed OS after LM diagnosis based on HR status (8.3 months in both groups; *P* = .434) or in patients with de novo vs recurrent breast cancer (8.9 vs 6.4 months; *P* = .578). Patients with primary invasive lobular cancer had a shorter OS after LM diagnosis than those with invasive ductal subtypes (1.7 vs 8.3 months; *P* = .676), but the difference was not statistically significant. Among clinical parameters evaluated at time of LM diagnosis, a poorer Karnofsky performance status (<70) (*P* = .015) and presence of neurological symptoms at the time of LM diagnosis (*P* = .011) were significantly associated with a significantly higher risk of death in univariate analysis. Among prognostic indices, two (SS‐BM score A, *P* = .001; Index score C or D, *P* < .001) were significantly associated with a significantly higher risk of death in univariate analysis. Moreover, patient diagnosed with LM prior to 2010 showed a worse prognosis as compared to patients diagnosed in more recent years (*P* = .036).

**TABLE 3:** Factors influencing OS from time of LM diagnosis (univariate analysis)

			Univariate analysis	
Variable		Median OS (mo); 95% CI	Hazard ratio (95% CI)	*P* value
HR status	HR+	8.3 (5.8‐10.9)	1	.498
	HR−	8.3 (0‐17.8)	1.18 (0.73‐1.91)	
Age	≥60 years	8.3 (1.8‐14.9)	1	.072
	<60 years	6.4 (0‐14.3)	1.71 (0.98‐2.97)	
Year of diagnosis	≥2010	8.3 (3.5‐13.2)	1	.036
	<2010	2.4 (0.2‐4.7)	2.22 (1.05‐4.70)	
KPS	≥70	12.6 (6.1‐19.1)	1	.015
	<70	2.9 (0.7‐5.1)	1.81 (1.12‐2.93)	
B‐GPA score	3‐4	9.5 (5.8‐13.2)	1	.055
	1‐2	2.7 (0–6)	1.65 (0.99‐2.77)	
SS‐BM score	B‐C	12.9 (6.7‐19.1)	1	.001
	A	2.3 (1‐3.5)	2.38 (1.47‐3.84)	
INDEX score	A‐B	14.2 (4.5‐24)	1	<.001
	C‐D	2.6 (1.9‐3.3)	2.42 (1.49‐3.91)	
Neurologic symptoms	No	20.4 (−/−)	1	.011
	Yes	6.6 (2.5‐10.6)	3.29 (1.31‐8.24)	
Extracranial disease under control at LM diagnosis	Yes	8.3 (3.1‐13.6)	1	.440
	No	8.8 (4.1‐13.6)	0.82 (0.49‐1.37)	
Brain metastases associated with LM	No	8.3 (5.6‐15.4)	1	.707
	Yes	6.9 (3.9‐9.9)	1.10 (0.67‐1.80)	
MRI findings	Focal	8.3 (8.3‐8.4)	1	.790
	Diffuse	5.2 (0.7‐9.8)	1.08 (0.60‐1.95)	
LM in the neuro‐axis	No	6.4 (0‐13.3)	1	.642
	Yes	4.4 (1.2‐7.6)	1.19 (0.57‐2.46)	
Hydrocephalus	No	8.3 (5.6–11)	1	.425
	Yes	2.6 (1.1‐4.1)	1.46 (0.58‐3.68)	
Chemotherapy after LM diagnosis	Yes	13.2 (5.1‐21.2)	1	.024
	No	3.8 (0‐8.2)	1.74 (1.01‐2.81)	
Trastuzumab ± pertuzumab	Yes	8.9 (2.5‐15.3)	1	.013
	No	2.8 (1.3‐4.3)	2.04 (1.16‐3.58)	
TDM–1 therapy after LM diagnosis	Yes	8.3 (0.5‐16.2)	1	.221
	No	6.4 (4.4‐12.2)	1.35 (0.84‐2.18)	
Lapatinib after LM diagnosis	Yes	7 (3.1‐10.8)	1	.487
	No	8.3 (0.7‐16)	0.84 (0.52‐1.37)	
Intrathecal chemotherapy after LM diagnosis	Yes	8.3 (6.9‐9.8)	1	.342
	No	6.6 (2.9‐10.2)	1.28 (0.76‐2.15)	
Radiation therapy after LM diagnosis	Yes	12.6 (6.4‐18.7)	1	.066
	No	2.4 (1.9‐2.9)	1.62 (0.97‐2.71)	

Abbreviations: BC, breast cancer; B‐GPA, breast‐specific graded prognostic assessment; CI, confidence interval; HER2, human epidermal growth receptor 2; HR, hormone receptor; INDEX score, prognostic index for patients with leptomeningeal carcinomatosis; KPS, Karnofsky performance status; LM, leptomeningeal metastases; MRI, magnetic resonance imaging; n, number; OS, overall survival; SS‐BM, simple survival score for patients with brain metastases; TDM‐1, trastuzumab‐emtansine.

The impact of treatment received after LM diagnosis on OS was also investigated using univariate Cox regression (Table 3). Treatment modalities significantly associated with OS from LM diagnosis were as follows: chemotherapy (OS difference 9.4 months, *P* = .024) and receiving monoclonal antibodies (trastuzumab ± pertuzumab; OS difference 6.1 months; *P* = .013). Patients treated with TDM‐1 (OS difference 1.9 months, *P* = .221), intrathecal chemotherapy (OS difference 1.7 months, *P* = .342), or RT after LM diagnosis (OS difference 10.2 months, *P* = .066) showed a longer median OS from time of LM diagnosis, but the difference was not statistically significant. Impact of receiving (any) HER2 targeted treatment could not be assessed as only a limited number of patients did not receive HER2 targeted treatment after LM diagnosis (n = 3). Patients who received multimodality therapy, including targeted therapy, chemotherapy, and RT (N = 30; 36.6%), had a longer OS (15.4 months, 95% CI 5.5‐25.4) compared to all other patients (N = 52, 63.4%) who received only two modalities of systemic/local treatment (3.8 months, 95% CI 5.6‐11.0). The difference was statistically significant (*P* = .040). Kaplan‐Meier survival curves are presented in Figure 2.

The seven clinical variables (excluding prognostic scores) associated with OS with *P*‐values <.10 at univariate analysis were included in the multivariate analysis. At multivariate analysis, only the presence of neurological symptoms at the time of LM diagnosis (hazard ratio 3.32, 95% CI 1.26‐8.73; *P* = .015) and not receiving RT after LM diagnosis (hazard ratio 2.02, 95% CI 1.09‐3.72; *P* = .024) were confirmed to be statistically significantly and independently associated with worse OS. Conversely, younger age <60 years (hazard ratio 1.05, 95% CI 0.49‐2.23; *P* = .897), LM diagnosis before 2010 (hazard ratio 2.41, 95% CI 0.88‐6.63; *P* = .088), Karnofsky performance status <70 (hazard ratio 1.63, 95% CI 0.97‐2.73; *P* = .064), not receiving chemotherapy (hazard ratio 1.68, 95% CI 0.71‐3.99; *P* = .239) or monoclonal antibodies trastuzumab and/or pertuzumab (hazard ratio 1.87, 95% CI 0.96‐3.72; *P* = .063) following LM diagnosis were not significantly associated with worse OS.

## 4 DISCUSSION

The treatment landscape of HER2+ metastatic breast cancer has significantly changed over the last decade. Even if increasing attention is given to the specificities of patients with CNS involvement and several anti‐HER2 targeted agents have demonstrated antitumor activity in the brain,^20^, ^21^, ^22^, ^23^, ^24^, ^25^ still little is known regarding the characteristics and clinical outcomes of patients with HER2‐positive breast cancer and LM.

We here report clinical and pathological characteristics, as well as survival outcomes, of 82 patients with HER2+ breast cancer and LM included in our real‐world multicentric retrospective cohort, which is one of the biggest cohorts published to date. In our study cohort, we observed a median OS from LM diagnosis of 8.3 months, which compares favorably to most previous studies,^10^, ^11^, ^14^, ^16^, ^32^ and 42% 1‐year OS rate, which is also higher than typically reported. Thanks to the availability of multiple effective anti‐HER2 treatments, the median OS of patients with metastatic HER2+ breast cancer has been greatly extended in recent years.^33^ However, patients with advanced HER2+ breast cancer patients and brain metastases (excluding patients with LM) have a lower OS than those without (26.3‐30 vs 38‐44.6 months), indicating that despite advances in targeted therapy, patients with CNS involvement continue to have a worse prognosis.^34^, ^35^ Patients with LM appear to have an even worse prognosis in this context. In fact, according to recent retrospective research, the median OS of patients with breast cancer diagnosed with LM is poor and ranges from 1.8 to 5.4 months.^13^, ^14^, ^15^, ^16^, ^17^, ^36^ Patients with HER2+ tumors and LM have a slightly longer median OS than patients with HER2‐negative breast cancer, reaching 7.0‐8.4 months. Clinical factors reported by previous studies to be associated with longer OS are patient's performance status and the use of systemic chemotherapy or anti‐HER2 targeted treatments.^13^, ^14^ Compared to previous studies (Table 4),^10^, ^11^, ^14^, ^16^, ^32^ patients in our cohort were older at the time of LM diagnosis, more patients had HR‐positive primary breast cancer, more patients received anti‐HER2 targeted therapy and fewer patients underwent RT and IT procedures.

**TABLE 4:** Studies evaluating OS in patients with HER2+ breast cancer and LM

	Niwinska 2013^11^	Abouharb 2014^16^	Morikawa 2017^14^	Zagouri 2020^29^ ^,^ ^a^	Carausu 2021^10^	Current study
Number of patients	22	56	83	54	47	82
Years of treatment	1999‐2009	1997‐2012	1998‐2013	2001‐2018	2008‐2016	2005‐2020
HR positivity, rate	36.3%	11%	32.5%	50.9%	NA	59.8%
Time from MBC to LM diagnosis (mo)	NA	9.8	22.6	NA	NA	21.1
Median age at LM diagnosis (y)	52	50	54	51	52	64
Percentage of patients with KPS ≥ 70	41	NA	70	25.9	29.9	46.3
Systemic chemotherapy rate (%)	64	59	60	NA	NA	50
Targeted therapy rate (%)	7	51	NA	NA	NA	96.3
RT rate (%)	80	75	64	86.1	12.5	58
Intrathecal therapy rate (%)	79	52	34	100^b^	100	30.5
One‐year survival rate	16%	21%	20%	NA	25%	42%
Median OS (mo, 95% CI)	4.6 (2‐7.3)	4.4 (NA)	5.2 (3.2‐7.8)^c^	13.2 (NA)^d^	5.6 (2.9‐11.6)^d^	8.3 (5.7‐11)

Abbreviations: CI, confidence interval; HER2, human epidermal growth receptor 2; HR, hormonal receptor; IT, intrathecal therapy; KPS, Karnofsky performance status; MBC, metastatic breast cancer; NA, not available; OS, overall survival; RT, any radiation therapy (including whole brain radiation therapy, focal brain therapy or spine radiotherapy).

^a^ All presented data were collected at the initiation of IT trastuzumab.

^b^ All patients received IT trastuzumab.

^c^ 3.3 months (2.4‐5.0) before year 2005 and 7.0 months (3.7‐12.2) after year 2005.

^d^ Overall survival was calculated from the date of IT.

Following LM diagnosis, the majority of patients in our study cohort received multimodality treatment, which included several systemic treatment approaches, IT, and RT. At univariate analysis, patients who were treated after 2010 (which coincides with the introduction of several anti‐HER2 therapies into clinical practice), or had better Karnofsky performance status, were free of neurological symptoms, had higher scores on the SS‐BM and INDEX prognostic indices, received chemotherapy or monoclonal antibodies (trastuzumab with or without pertuzumab) after LM diagnosis, had a statistically significantly longer OS. At multivariate analysis, absence of neurological symptoms at time of LM presentation, which is consistent with previous reports,^10^, ^28^, ^36^, ^37^, ^38^, ^39^ and undergoing RT remained statistically significant predictors of better survival.

In our study cohort, 62.2% of patients were diagnosed with brain metastases prior to or concurrently with LM, and 18.1% underwent brain surgery, before the development of LM, as a direct consequence of brain metastases. At the time of LM diagnosis, 70.7% of patients in our cohort received RT, the majority of which was whole brain RT, and 22.4% of them were previously treated with RT for either brain parenchymal metastases or due to LM. Patients who received the second RT course might have tumor cells that responded better to various therapies, and they lived long enough to be considered for reirradiation due to CNS symptoms or disease progression in the brain. Though RT has the potential to improve quality of life in the short term and is used for the relief of neurological symptoms, the treatment of bulky disease, and the normalization of cerebrospinal fluid flow, data on the effect of RT on survival outcomes are inconsistent,^18^, ^40^ and RT can contribute to neurocognitive function deficits in a longer‐term follow‐up.^10^, ^28^ Trastuzumab levels in CSF fluid have been shown to increase when the blood‐brain barrier is compromised by local disease (such as LM) or following RT due to increased blood‐brain barrier permeability.^41^ Despite this, trastuzumab levels in the cerebrospinal fluid appear to be low when compared to the therapeutic serum concentration.^42^ Aside from the blood‐brain barrier, which may make chemotherapeutics and anti‐HER2 therapy ineffective against brain lesions, HER2+ breast cancer tumor cells can develop molecular mechanisms to avoid anti‐HER2 therapy in the brain.^43^ Nevertheless, in a recent metaanalysis, researchers reported a trend toward improved CNS progression‐free survival in patients who had previously undergone RT or neurosurgery to treat brain metastases.^32^

In our study cohort, about one‐third of patients received IT either alone or in combination with systemic chemotherapy and trastuzumab. Only two patients received trastuzumab intrathecally. When compared to patients who did not receive IT, the observed gain in OS was small and not statistically significant. In a recent retrospective analysis of a large contemporary cohort of 312 patients with metastatic breast cancer and LM treated with IT, the authors reported a median OS of 4.5 months and a 1‐year OS rate of 25% for the entire cohort, regardless of HER2 status.^10^ In the same study, concomitant systemic therapy was associated with better OS. In comparison to our cohort, patients in the aforementioned study were younger at time of LM diagnosis and were more frequently treated with RT. Although the addition of IT is not considered standard therapy because it has not demonstrated quality of life improvements or a meaningful OS advantage, it can be considered for some patients after a prognostic evaluation and multidisciplinary team discussion.^44^ IT trastuzumab was being studied in patients with LM from HER2+ breast cancer, either alone or in combination with other therapies. Even when combined with systemic therapy, IT trastuzumab is well tolerated when injected intrathecally. According to Zagouri et al metaanalysis, following IT administration of trastuzumab, 55.0% of cases showed a significant improvement, 27.5% experienced disease stabilization, and 17.5% experienced disease progression.^32^ This metanalysis of all currently available data revealed that IT trastuzumab appears to be a promising treatment option in patients with LM from HER2+ breast cancer. The results of this metaanalysis showed that the OS in patients treated with IT trastuzumab was 13.2 months from the start of IT trastuzumab.^32^ Nonetheless, because this metaanalysis incorporates multiple patient case reports, potential bias in patient selection cannot be ruled out.

Approximately two‐thirds of patients in our study had a preexisting diagnosis of metastatic breast cancer at time of LM diagnosis, which is similar to previous reported cohorts.^10^, ^36^ We found only a minor and nonsignificant difference in median OS (2.5 months) following LM diagnosis in patients with de novo metastatic breast cancer compared to recurrent metastatic breast cancer, implying that both groups of patients seem to have a similar prognosis.

As almost all patients included in our study received at least one line of anti‐HER2 targeted therapy, we were unable to assess the impact of targeted treatment on survival for patients receiving anti‐HER2 therapy (including trastuzumab, TDM‐1, and lapatinib) vs those who did not. Nevertheless, in a univariate analysis, the receipt of trastuzumab (with or without pertuzumab) resulted in significantly improved OS. More than a third of patients diagnosed with LM in our study cohort received TDM‐1 or lapatinib, respectively. There was a 1.9‐month difference in survival between those who received TDM‐1 therapy and those who did not, but the difference was not statistically significant. New HER2‐targeted agents have shown extremely promising antitumor activity in patients with HER2+ breast cancer and brain metastases.^20^, ^21^, ^22^, ^23^, ^24^, ^25^, ^45^ However, little is known about the efficacy of these novel drugs in patients with LM. Further research is being conducted to test the survival outcome of patients with advanced HER2+ breast cancer and CNS involvement—including patients with LM—with new HER2‐targeted treatments with or without RT (ClinicalTrials.gov identifiers NCT04420598, NCT04856475, NCT03661424, NCT04457596) and MRI screening (ClinicalTrials.gov identifier NCT04030507).

Our study has several strengths: we analyzed one of the largest studied to date, homogeneous cohort of patients with HER2+ breast cancer diagnosed with LM across three European countries; in addition, patients included were diagnosed and treated in relatively recent years, when several HER2‐targeted agents were already available. Nevertheless, data reported in our study was collected retrospectively and might therefore be subject to potential biases linked to its retrospective nature. Moreover, patients were treated by clinical practice, therefore patients who received treatment may have had better Karnofsky performance status and less advanced disease, resulting in a survival bias.

To summarize, LM is a fatal complication of HER2‐positive breast cancer for which the optimal systemic or local treatment still remains to be defined. In our cohort of patients with HER2‐positive breast cancer we observed a median OS of 8.3 months from LM diagnosis, with a significant proportion of patients alive at 1 and 2 years after diagnosis (42% and 21%, respectively), which is considerably longer than reported by most previous studies. In the univariate analysis, absence of neurological symptoms, good performance status at time of LM diagnosis, receipt of chemotherapy or trastuzumab (with or without pertuzumab), were all associated with longer OS. At a multivariate model, both absence of neurological symptoms and having received RT were associated with longer OS. However, due to the study's retrospective nature, it is impossible to assess the real benefit of each treatment modality as some patients might have received certain therapies because they lived longer and, for example, got local treatment after developing symptoms or because their condition was so good that systemic treatment was possible. The patient's performance status, on the other hand, is a crucial modifying element for therapy decisions. Moreover, the impact of overall HER2‐targeted treatment was not assessable, as almost all patients in our study cohort received at least one line of targeted therapy.

In conclusion, treatment of patients with HER2+ breast cancer and LM remains challenging, and until the results of prospective studies are available, a careful evaluation of all possible treatments and an individual approach for each patient is required. Being without neurological symptoms and receiving RT at the time of LM diagnosis were related with a longer OS in our study cohort. Patients with LM who underwent multimodality therapy, which included targeted therapy, chemotherapy, and RT to the LM sites, had a higher rate of OS.

## AUTHOR CONTRIBUTIONS

The work reported in the paper has been performed by the authors, unless clearly specified in the text: Conceptualization: Ivica Ratosa, Gaia Griguolo, Amélie Darlix, Stéphane Pouderoux, William Jacot; Data curation: Ivica Ratosa, Nika Dobnikar, Tanja Znidaric, Michele Bottosso, Amélie Darlix, Léa Sinoquet; Formal Analysis: Ivica Ratosa, Nika Dobnikar, Tanja Znidaric, Gaia Griguolo, Amélie Darlix, Stéphane Pouderoux, William Jacot; Funding acquisition: none; Investigation: none; Methodology: Ivica Ratosa, Gaia Griguolo, Amélie Darlix, William Jacot; Project administration: Ivica Ratosa, Nika Dobnikar, Domen Ribnikar, Tanja Znidaric, Gaia Griguolo, Amélie Darlix; Resources: Ivica Ratosa, Nika Dobnikar, Domen Ribnikar, Tanja Znidaric, Gaia Griguolo, Amélie Darlix, William Jacot; Software: none; Supervision: Ivica Ratosa, Gaia Griguolo, Amélie Darlix, William Jacot; Validation: Ivica Ratosa, Domen Ribnikar, Tanja Znidaric, Gaia Griguolo, Amélie Darlix, William Jacot; Visualization: none; Writing—original draft: Ivica Ratosa, Nika Dobnikar; Writing—review & editing: Ivica Ratosa, Nika Dobnikar, Michele Bottosso, Maria Vittoria Dieci, William Jacot, Stéphane Pouderoux, Domen Ribnikar, Léa Sinoquet, Valentina Guarneri, Tanja Znidaric, Amélie Darlix, Gaia Griguolo; All authors read, provided feedback and approved the final protocol and manuscript.

## FUNDING INFORMATION

The authors acknowledge grants from: Istituto Oncologico Veneto projects L04P11 (to Valentina Guarneri), L02P03 (to Maria Vittoria Dieci), L04P25 (to Maria Vittoria Dieci); University of Padova—Department of Surgery, Oncology and Gastroenterology BIRD 2020 and BIRD 2021 (to Valentina Guarneri, Maria Vittoria Dieci, Gaia Griguolo); 2019 Conquer Cancer Foundation of ASCO/Shanken Family Foundation Young Investigator Award (to Gaia Griguolo); Fondazione AIRC under 5 per mille 2019—ID. 22759 program—G.L. Valentina Guarneri.

## CONFLICT OF INTEREST

Ivica Ratosa, Nika Dobnikar, Michele Bottosso, Amélie Darlix, William Jacot, Stéphane Pouderoux, Domen Ribnikar, Léa Sinoquet and Tanja Znidaric report no conflict of interest. Maria Vittoria Dieci reports personal fees from Eli Lilly, MSD, Exact Sciences, Novartis, Pfizer, Seagen, outside the submitted work. Valentina Guarneri reports personal fees from Eli Lilly, Novartis, Roche, MSD, Gilead, Eisai (Advisory Board), and from Eli Lilly, Novartis, GSK, Amgen (Speakers' Bureau) outside the submitted work. Gaia Griguolo reports personal fees from Eli Lilly and Novartis (invited speaker), Gilead (Advisory Board) and from Novartis, Pfizer, Amgen, Daiichi Sankyo (Travel Support) outside the submitted work.

## ETHICS STATEMENT

All procedures performed in studies involving human participants were in accordance with the ethical standards of the institutional and/or national research committees and with the 1964 Helsinki declaration and its later amendments or comparable ethical standards. Our study was approved by the institutional review board and ethical committee (approval number ERID‐KSOPKR‐0074/2020). All study participants provided informed consent for the use of their data for retrospective research purposes, if required by local regulations.

## Supporting information


**Appendix S1** Supporting Information

## Data Availability

Access to the de‐identified data on a secure web application REDCap (Research Electronic Data Capture) platform can be made available to qualified researchers upon reasonable request. Further information is available from the corresponding author upon request.
