# Supplementary material for: Leptomeningeal metastases in patients with human epidermal growth factor receptor 2 positive breast cancer: Real‐world data from a multicentric European cohort
Source: Int J Cancer. 2022 Jun 25;151(8):1355–66. doi: 10.1002/ijc.34135 (PMC9540903; doi:10.1002/ijc.34135)
Supplement: Supplementary file 1 — Appendix S1 Supporting Information [file IJC-151-1355-s001.pdf]

Leptomeningeal metastases in patients with human epidermal growth factor receptor 2 positive breast cancer: real-world data from a multicentric European cohort

Ivica Ratosa, Nika Dobnikar, Michele Bottosso, Maria Vittoria Dieci, William Jacot, Stéphane Pouderoux, Domen Ribnikar, Léa Sinoquet, Valentina Guarneri, Tanja Znidaric, Amélie Darlix, Gaia Griguolo

Table of Contents

1. Supplementary Materials and Methods 2

2. Supplementary tables 4

1. Supplementary Materials and Methods

Identification of breast cancer subtypes

Routine diagnostic pathology reports were used to assess the oestrogen receptor (ER), progesterone receptor (PR), and Human epidermal growth receptor 2 (HER2) status of primary breast cancer tumours or metastatic disease at the time of diagnosis and were evaluated according to then valid American Society of Clinical Oncology/College of American Pathologists (ASCO/CAP) recommendations. Hormone receptor–positive disease was classified as ER+ and/or PR+ disease with a cut off value of 1 %. The methods for detecting ER, PR, and HER2 varied between years 2005 and 2020.

The detection methods for ER or PR

For the detection of **ER**, immunohistochemistry (IHC) methods were as follows:

- **Years 2005 – 2011:** Antibody clone SP1 (Estrogen Receptor, RM-9101-S, 1:25, NeoMarkers ThermoFisher Scientific, Kalamazoo MI), staining platform Autostainer 480 (LabVisionTM, Denmark), detection DAKO REALTM EnVisionTM Detection System (K5007, RTU, DAKO, Golstrup, DK) or SP1 (790-4325, Ventana Medical System, Tucson AZ, pre-diluted) or Clone 6F11 (Novocastra™).

- **Years 2011 – 2014**: antibody clone SP1 (Estrogen Receptor, RM-9101-S, 1:25, NeoMarkers ThermoFisher Scientific, Kalamazoo MI), staining platform Benchmark XT (Ventana medical system, Tuscon, AZ), detection UltraView universal DAB Detection Kit (760-500, RTU, Ventana medical system, Tuscon, AZ) or 6F11 (PA0153, Leica Biosystems Newcastle, Newcastle UK,pre-diluted) or Clone EP1 (DAKO, diluted).

- **Years 2014 – 2020**: Antibody clone SP1 (Estrogen Receptor, RM-9101-S,1:200, NeoMarkers ThermoFisher Scientific, Kalamazoo MI), staining platform Benchmark Ultra or Benchmark XT (both Ventana medical systems, Tuscon, AZ), detection OptiView DAB IHC Detection Kit (760-700, RTU, Ventana medical systems, Tuscon, AZ) or 6F11 (PA0153, Leica Biosystems Newcastle, Newcastle UK, pre-diluted) or Clone EP1 (DAKO, Golstrup, DK, diluted).

For the detection of **PR**, IHC methods were as follows:

- **Years 2005 – 2011**: Antibody clone PgR636 (Progesterone receptor, M3569, 1:50, DAKO, Golstrup DK), staining platform Autostainer 480 (LabVision™, Denmark), detection DAKO REAL™ EnVision™ Detection System (K5007, RTU, DAKO, Golstrup, DK) or 1E2 (790-4296, Ventana Medical System, Tucson AZ, pre-diluted).

- **Years 2011 – 2013**: antibody clone 1E2 (anti-PR 1E2 Rabbit Monoclonal primary Antibody, 790-2223, RTU, Ventana medical systems, Tuscon, AZ), staining platform Benchmark XT (Ventana medical systems, Tuscon, AZ), detection UltraView universal DAB Detection Kit (760-500, RTU, Ventana medical systems, Tuscon, AZ) or 16 (PA0322, Leica Biosystems Newcastle, Newcastle UK, 1:100).

- **Since 2014**: Antibody clone 16 (Novocastra™ Liquid Mouse Monoclonal Antibody Progesterone Receptor, NCL-L-PGR-312, 1:800, Leica Biosystems, NewCastle, UK), staining

platform Autostainer 480 (LabVision™, Denmark), detection EnVision™ FLEX High pH (K8000, RTU, DAKO, Golstrup, DK) or 16 (PA0322, Leica Biosystems Newcastle, Newcastle UK, 1:100) or Clone PgR636 (DAKO, Golstrup, DK, diluted).

The detection of HER2 status

By IHC:

- **Years 2005 – 2010**: Polyclonal antibody (HercepTestTM for TechmateTM Instruments, K5206, RTU, DAKO, Golstrup, DK), staining platform Autostainer 480 (LabVisionTM, Denmark), detection DAKO REALTM EnVisionTM (K5007, RTU, DAKO, Golstrup, DK).

- **Years 2011 – 2020**: Antibody clone 4B5 (VENTANA anti-HER2/neu (4B5) Rabbit Monoclonal Primary Antibody; 790-2991, RTU, Ventana medical systems, Tuscon, AZ), staining platform Benchmark XT (Ventana medical systems, Tuscon, AZ), detection UltraView universal DAB Detection Kit (760-500; RTU, Ventana medical systems, Tuscon, AZ) or antibody A0485 (DAKO, Golstrup, DK).

By fluorescent in situ hybridization (FISH):

- The detection of HER2 status by FISH was performed in all cases regardless of IHC score and the same method was used in **years 2005 – 2020**: Path Vysion HER2 DNA probe kit II (06N46-036, RTU, Abbott Vysis, Abbott Park, IL).

2. Supplementary tables

Supplemental table 1: Systemic treatment received for metastatic breast cancer before LM diagnosis.

**Treatment modality**	All patients<br>(n=82, 100%)	HR+<br>(n=49, 100%)	HR-<br>(n=33, 100%)
**Endocrine therapy**			
0	53 (64.6)	22 (44.9)	33 (100)
1 line	23 (28.0)	21 (42.9)	0
≥2 lines	6 (7.3)	5 (12.2)	0
**Chemotherapy**			
0	25 (30.5)	16 (32.7)	9 (10.9)
1 line	25 (30.5)	14 (28.6)	11 (13.4)
≥2 lines	32 (39.0)	18 (21.9)	13 15.6)
**Any anti-HER2 therapy***			
0	17 (23)	11 (13.4)	6 (7.3)
1 line	35 (47.3)	21 (25.6)	14 (17.1)
≥2 lines	22 (19.9)	11 (13.4)	11 (13.4)
Missing data	8 (9.8)		

* Anti-HER2 therapy includes trastuzumab, trastuzumab emtansine, lapatinib.

Abbreviations: n = Number; LM = Leptomeningeal metastases; HR = Hormonal receptors.
